# Model-based somapacitan dosing and IGF-I response in children born SGA or with ISS, Noonan or Turner syndromes

**DOI:** 10.1210/jendso/bvag124

**Published:** 2026-06-04

**Authors:** Julie Desrochers, Philippe Backeljauw, Michael Højby, Rasmus Juul Kildemoes, Agnès Linglart, Jun Mori, Rory Forseth Leisegang

**Affiliations:** Medical & Translational Sciences, Novo Nordisk A/S, Søborg 2860, Denmark; Division of Diabetes and Endocrinology, Cincinnati Children's Hospital Medical Center, University of Cincinnati College of Medicine, Cincinnati, OH 45229, USA; Medical & Translational Sciences, Novo Nordisk A/S, Søborg 2860, Denmark; Medical & Translational Sciences, Novo Nordisk A/S, Søborg 2860, Denmark; Université Paris-Saclay, AP-HP, INSERM, Service d'Endocrinologie et diabète de l'enfant, Hôpital Bicêtre Paris Saclay, F-94270 Le Kremlin-Bicêtre, France; Division of Pediatric Endocrinology, Metabolism and Nephrology, Children's Medical Center, Osaka City General Hospital, Osaka 534-0021, Japan; Medical & Translational Sciences, Novo Nordisk A/S, Søborg 2860, Denmark

**Keywords:** somapacitan dosing, IGF-I monitoring, small for gestational age, Noonan syndrome, idiopathic short stature, Turner syndrome

## Abstract

**Context:**

The weekly insulin-like growth factor I (IGF-I) profile for children and adolescents treated with long-acting growth hormone differs from that of daily growth hormone (GH).

**Objective:**

The objective of this study is to provide guidance on the use of once-weekly somapacitan and IGF-I monitoring in prepubertal short children and adolescents born small for gestational age (SGA) or with idiopathic short stature (ISS), Noonan syndrome (NS), or Turner syndrome (TS).

**Methods:**

Modeling, including population pharmacokinetic/pharmacodynamic (PK/PD) modeling, were utilized, analyzing IGF-I data from 4 clinical studies, including 2 phase 3 trials (REAL8: NCT05330325; REAL9: NCT05723835) involving children and adolescents born SGA (*N* = 80), ISS (*N* = 69), NS (*N* = 62), and TS (*N* = 79), along with additional data from phase 2 (REAL5: NCT03878446, *N* = 59) and phase 1 trials (NCT01973244, *N* = 24).

**Results:**

Relationships between somapacitan dose, exposure, baseline IGF-I SD score (SDS), and height velocity (HV) were established in children born SGA, with similar responses anticipated for those with ISS, NS, or TS. A linear model enabled the estimation of average weekly IGF-I exposure from a single sample collected during the somapacitan dosing interval. IGF-I SDS simulations support flexible dosing changes while maintaining a minimum of 4 days between doses.

**Conclusion:**

Somapacitan 0.24 mg/kg/week produced similar IGF-I SDS changes and height velocity increases as daily GH in prepubertal short children and adolescents born SGA or with ISS, NS, or TS. The results support that the guidance already established for GHD regarding somapacitan initiation, dosing flexibility, and IGF-I monitoring remain appropriate for prepubertal short children and adolescents born SGA or with ISS, NS, or TS.

In recent years, long-acting growth hormone (LAGH) formulations have gained approval in various regions for the treatment of growth hormone deficiency (GHD). Among these formulations, once-weekly somapacitan (Sogroya®, Novo Nordisk A/S) is currently undergoing clinical development for the treatment of short stature in children and adolescents born small for gestational age (SGA) or with idiopathic short stature (ISS), Noonan syndrome (NS), or Turner syndrome (TS). The motivation for providing growth hormone (GH) is consistent across all 4 indications, namely, to address short stature and/or growth failure resulting from either environmental and/or genetic factors (SGA), an undetermined cause (ISS), or specific genetic modifications (NS and TS).

GH stimulates insulin-like growth factor I (IGF-I) release, which is an efficacy biomarker in pediatric GHD and non-GHD [1], whereas IGF-I monitoring is further helpful to evaluate and monitor adherence with GH treatment [2-5]. Daily GH injections fail to replicate endogenous IGF-I release patterns, yet IGF-I concentrations remain relatively stable [6]. With LAGH treatment, distinct pharmacokinetics and IGF-I profile over the weekly dosing interval are noted: a higher peak plasma concentration (*C*_max_) in the first days after dosing that decreases to lower concentrations on day 7 (trough value) just before the next dose [7]. This profile necessitates a deeper understanding of the pharmacokinetic (PK) and pharmacodynamic (PD) dynamics to ensure optimal blood sampling timing for monitoring IGF-I concentrations in individuals treated with LAGHs [8, 9].

Two ongoing phase 3 studies in prepubertal short children (REAL8: NCT05330325) and adolescents (REAL9: NCT05723835) aim to confirm the efficacy and safety of once-weekly somapacitan. This study presents dosing and IGF-I monitoring based on PK/PD modeling analyses, which leverages data from these 2 phase 3 studies, supplemented with PK and PD data from a previously published phase 2 study in SGA (REAL5: NCT03878446) [10] and a phase 1 study in GHD. PK/PD models were developed to characterize somapacitan and IGF-I profiles, predict dose-response, and support dosing day change calculations. Specifically, this study aims to provide treating physicians guidance for the initiation and use of somapacitan and the monitoring of IGF-I in children and adolescents born SGA or with ISS, NS, or TS.

## Materials and methods

### Clinical study data

The analyses were conducted based on data from participants with short stature enrolled in 4 [4] clinical studies: (1) a multinational single-dose phase 1 study in previously GH-treated children with GHD (NCT01973244); (2) a global phase 2 dose-finding study in treatment-naïve children with SGA (NCT03878446, REAL5), (3) a global phase 3 study in treatment-naïve children with SGA, ISS, NS or TS (REAL8); and (4) a global phase 3 study in treatment-naïve adolescents with SGA, ISS, NS or TS (REAL9) (Table 1).

**Table 1:** Summary of clinical study designs

Study	NCT01973244	NCT03878446 (REAL5)	NCT05330325 (REAL8)	NCT05723835 (REAL9)
Clinical study design	Phase 1, randomized, active-controlled, multinational, dose-escalation study	Phase 3, randomized, active-controlled, multi-national, 5-arm, parallel group study	Phase 3, randomized, multi-national study to evaluate the non-inferiority of somapacitan vs Norditropin®	Phase 3, interventional, multi-national, multi-center, study
Study duration	28-35 days	26 weeks main + up to 260 weeks (extension)	52 weeks + 104 weeks safety extension + up to 117 weeks of extension (total up to 273 weeks)	26 weeks main + 130 weeks safety extension I + 91 weeks safety extension II (total up to 247 weeks)
Treatment regime	Somapacitan SD (0.02, 0.04, 0.08, 0.16 mg/kg) or Norditropin® (0.03 mg/kg/day for 7 days)	Somapacitan (0.16, 0.20, or 0.24 mg/kg/week) or Norditropin® FlexPro (0.035 or 0.067 mg/kg/day)	Somapacitan (0.24 mg/kg/week) vs Norditropin® daily (0.035, 0.050 or 0.067 mg/kg/day, depending on sub-study design for each indication)	Somapacitan (0.24 mg/kg/week) for 26 weeks, followed by extensions of 0.24 mg/kg/week
Participants enrolled	32 non-naïve pre-pubertal children with GHD	62 GH treatment naïve prepubertal children born SGA	GH treatment naïve children with short stature (SGA, TS, NS, ISS)	GH treatment naïve or non-naïve children with short stature (SGA, TS, NS, ISS)
Population	Global	Global	Global	Global
Comparator	Norditropin®	Norditropin®	Norditropin®	none
Participants randomized or included	Somapacitan: 24, Norditropin®: 8	Somapacitan: 37, Norditropin®: 24	SGA: 142; NS: 77; ISS: 88; TS:105	Included, not randomized: SGA: 12; NS: 13; ISS: 11; TS:11
Participants treated with somapacitan	24	37	SGA: 68; NS: 49; ISS: 58; TS:68	SGA: 12; NS: 13; ISS: 11; TS:11
Participants treated with daily GH	8	24	SGA: 72; NS: 28; ISS: 28; TS:37	0

Abbreviations: GH, growth hormone; ISS, idiopathic short stature; NS, Noonan syndrome; SGA, small for gestational age; TS, Turner syndrome.

The phase 1 study (NCT01973244) included 32 previously GH-treated children with GHD enrolled into 4 ascending somapacitan dose cohorts with a daily GH control group. Within each cohort, 6 children were randomized to receive a single s.c. dose of somapacitan of 0.02, 0.04, 0.08, or 0.16 mg/kg; 8 children were randomized to receive multiple s.c. doses of daily GH (0.03 mg/kg/day Norditropin®) for 7 days.

The phase 2 study (REAL5) included 62 prepubertal GH treatment-naïve children born SGA who were randomized in a 1:1:1:1 ratio for treatment with s.c. somapacitan (0.16, 0.20 or 0.24 mg/kg/week) or s.c. daily GH (0.035 or 0.067 mg/kg/day Norditropin®) for 26 weeks. All participants followed this dosing regimen for a 26-week main phase and a subsequent 26-week extension I Phase, totaling 52 weeks. After this, all participants were switched to receive somapacitan 0.24 mg/kg/week for a 208-week extension phase. This study formed the basis somapacitan chosen for the confirmatory phase 3 studies.

The first phase 3 study (REAL8) included GH treatment naïve children born SGA (*N* = 142) who were randomized in a 2:1:1 manner to receive either somapacitan 0.24 mg/kg/week, daily GH 0.035 mg/kg/day (Norditropin®) or 0.067 mg/kg/day (Norditropin®) for the first 52 weeks. Children with ISS (*N* = 77), NS (*N* = 88), and TS (*N* = 105) were randomized in a 2:1 manner to receive either somapacitan (0.24 mg/kg/week) or daily GH (0.050 mg/kg/day Norditropin®) for the first 52 weeks. After week 52, all children were allocated to receive 0.24 mg/kg/week somapacitan for a currently ongoing 104-week extension period.

The second phase 3 study (REAL9) included GH treatment naïve and non-naïve adolescents born SGA (*N* = 12) or with ISS (*N* = 11), NS (*N* = 13), or TS (*N* = 11) who received somapacitan 0.24 mg/kg/week for 26 weeks, followed by a 130-week extension phase currently ongoing. The data included in this paper cover up to 52 weeks of data for studies REAL8 and REAL9.

### Blood sampling and bioanalysis

REAL5, REAL8, and REAL9 included serial sparse blood samplings performed throughout the studies at selected time windows relative to dosing to cover baseline, peak, weekly average, and trough PK and IGF-I concentrations following once-weekly somapacitan dosing (before first dose, 0.5-2 days after dosing, 2-4 days after dosing and 4-6 days after dosing as well as pre-dosing before next dosing, respectively). In the phase 1 study, intensive sampling (14 samples per participant) was performed until 10 days after the single somapacitan dose to characterize the PK/PD profile. Dosing time was recorded in dosing diaries and used to calculate the actual time after dose for blood samples.

Somapacitan serum concentrations were measured using a validated somapacitan-specific Luminescent Oxygen Channeling Immunoassay developed by Novo Nordisk A/S (RRID: AB_231545, RRID: AB_3717418). The lower limit of quantification (LLOQ) was 0.5 ng/mL for all studies. IGF-I was analyzed at a central laboratory using commercially available assay kit (Immuno Diagnostic Systems immunoassay [ISYS] assay, Cat. # IS-3900, RRID: AB_2861357). LLOQ was 10 ng/mL. IGF-I values were analyzed on the ng/mL scale, and IGF-I standard deviation score (SDS) values were calculated using previously published reference data [11]. In-house assays were developed for anti-somapacitan antibodies (RRID: AB_3717418).

### Population PK/PD and exposure-response models

#### Population PK/PD analysis of somapacitan

The somapacitan population PK/PD models were based on previously published models developed from children with GHD [12, 13] and validated in SGA [10]. The approach consisted of adapting a previously reported structural model of somapacitan for GHD using clinical data from the patient population of interest (SGA, ISS, NS, and TS) and accounting for the variability and covariates in these populations. The model consisted of a non-linear PK model and an indirect response model of IGF-I. The structural elements of interest for the analysis were the relative bioavailability parameter (F_rel_), the baseline IGF-I production rate (*k*_in_), and the maximum somapacitan response of IGF-I (*E*_max_). Pre-specified covariates evaluated in the population PK/PD model development were sex, race/ethnicity, body weight, and population. Interindividual variability for the parameters of interest was assumed to be log-normal; residual error was log-normal for PK and proportional for IGF-I (on ng/mL scale). Dose–response analyses were evaluated in children born SGA, leveraging data from the phase 2 (REAL5) and phase 3 (REAL8) studies.

#### Population PK/PD analysis of daily GH

The daily GH (Norditropin®) population PD model was based on IGF-I data collected through week 52 in REAL5 and REAL8 studies. An indirect response model was estimated with primary parameters of interest, *k*_in_ and *E*_max_. The daily GH (Norditropin®) population PK model was a one-compartment model with linear absorption and a GH baseline parameter to account for endogenous GH. CL/F and GH baseline were estimated while the other PK model parameters were fixed to previously established values for daily GH [12]. The concentration corresponding to half-maximum stimulation of IGF-I production rate (EC_50_) and *K*_in_ were estimated. The drug-independent IGF-I turnover rate *k*_out_ was assumed to be the same for somapacitan, and *E*_max_ was fixed to the previously established value for daily GH [12].

#### Estimation of individual IGF-I SDS profiles and average

For each participant, the individual predicted weekly IGF-I profile at steady state, corresponding to their last visit (week 52 in most participants), was estimated using the final population PK/PD models for somapacitan and daily GH, respectively. IGF-I profiles were calculated on the ng/mL scale and converted to SDS. Weekly IGF-I average (IGF-I_avg_) SDS was derived as the area under the curve from individually estimated IGF-I SDS profiles divided by dosing interval.

#### Relationship between sample time after dose and average for somapacitan

Linear modeling was used to estimate at different sampling times after dose (TAD), the difference between weekly IGF-I_avg_ SDS and IGF-I_TAD_ SDS for subjects who received 0.24 mg/kg somapacitan (week 0-52) in the studies REAL5, REAL8, and REAL9. IGF-I SDS was analyzed on the normal scale. Results were approximated into a correction parameter to calculate IGF-I_avg_ from a sample collected within daily (24-hour) intervals for ease of use. A forest plot was created to visualize the mean differences with a 90% CI between the adjustments determined previously for the GHD indication and the adjustments estimated for children and adolescents born SGA or with ISS, NS, or TS.

#### Dose-IGF-I analysis and exposure–response analysis on HV

The relationship between somapacitan dose (mg/kg) and individual predicted change from baseline in IGF-I_avg_ SDS at steady state (last visit) from participants born SGA in REAL8 was investigated with a linear model with baseline IGF-I SDS as a covariate.

Exposure–response analyses were conducted based on week 52 data, using a log-linear relationship between somapacitan exposure and HV, and a linear relationship between the change from baseline IGF-I_avg_ SDS and HV. Included covariates were sex, age group, height SDS group, baseline IGF-I SDS and baseline HV, and region, with a sex by age group by region interaction.

#### Estimation and software

Population PK/PD model parameters for somapacitan were estimated by simultaneously fitting PK and IGF-I data using the NONMEM software. For daily GH, a sequential approach was used to estimate PD and PK model parameters. Data below LLOQ were set to LLOQ/2. Full population PK/PD models were constructed with all investigated covariates included [14]. The 90% CI for each covariate effect was estimated by bootstrapping. Covariate-parameter relationships with a 90% CI that overlapped “no effect” (0 and 1 for continuous and categorical covariates, respectively) were not included in the final PK/PD models. NONMEM v7.5 (ICON Development Solutions, Ellicott City, MD, USA) and R version 4.4.1 (R Foundation for Statistical Computing, Vienna, Austria) with PsN version 5.31 (https://uupharmacometrics.github.io/PsN/index.html*, University of Uppsala)* were used for the population PK/PD and exposure-response modeling analyses.

## Results

### Data for PK/PD analyses

Baseline demographics and characteristics of the participants across all included studies and indications for somapacitan daily GH treatment are presented in the Supplementary Material—see Table S1 and Table S2, respectively [15].

### Population PK/PD modeling

The final somapacitan population PK/PD models successfully converged with the covariance step. The PK of somapacitan exhibited non-linear characteristics with a PK profile similar to those previously described for somapacitan in GHD [12]. Across all final PK models, body weight was the primary driver for exposures; the impact of sex and race was negligible. When compared to participants born SGA, somapacitan exposures were numerically higher in participants with NS, numerically lower in participants with TS, and comparable to exposure in ISS. Finally, following weight-based dosing, the analysis demonstrated that lower body weight was associated with slightly higher somapacitan exposure, while higher body weight resulted in slightly lower exposure compared to that of a reference individual.

**Figure 1:**
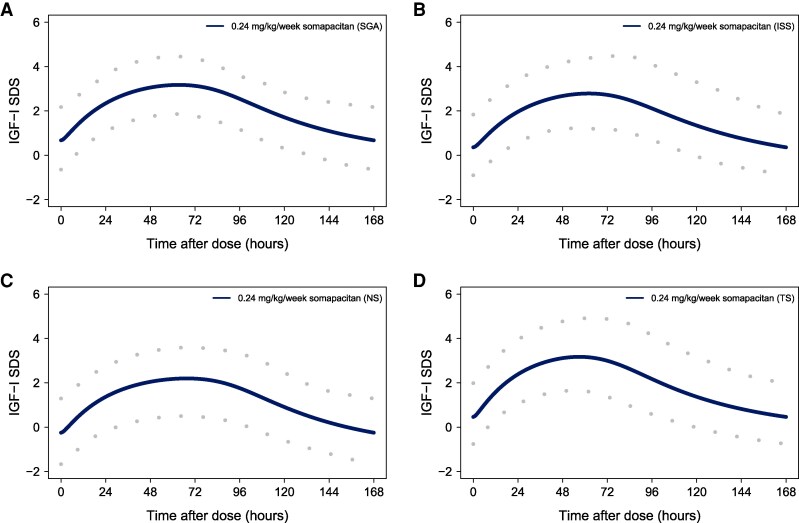
Weekly IGF-I SDS profile in prepubertal short children born SGA or with ISS, NS, or TS. Note: The solid blue lines represent the mean profile, and the dotted gray lines represent the 5th to 95th percentiles derived from the population PK/PD models. Abbreviations: GHD, growth hormone deficiency; IGF-I, insulin-like growth factor-I; ISS, idiopathic short stature; NS, Noonan syndrome; SDS, standard deviation score; SGA, small for gestational age; TS, Turner syndrome.

Across all final PD models, the covariate effects identified on *E*_max_ and kin indicate differences in IGF-I concentrations (ng/mL) between the groups at baseline and during treatment. However, these appeared to be correlated with age and sex, and no clinically meaningful covariate effects were found on IGF-I when evaluated as a change from baseline IGF-I_avg_ SDS. The IGF-I SDS range showed minor differences across indications. The mean IGF-I_avg_ SDS for each indication was respectively 2.17 for SGA, 1.81 for ISS, 1.13 for NS, and 1.93 for TS (Fig. 1).

### IGF-I evaluation following once-weekly somapacitan

**Figure 2:**
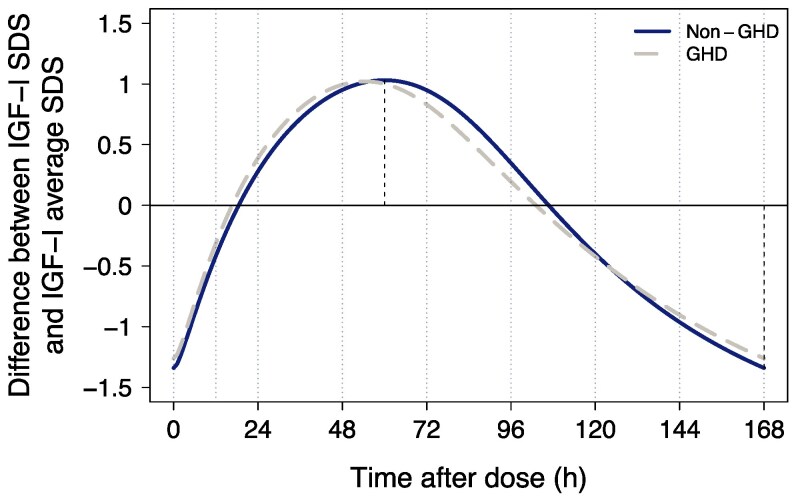
Difference between observed IGF-I SDS and IGF-I_avg_ SDS over the course of a week in children born SGA or with ISS, NS, or TS compared to children with GHD. Note: The dark blue curve indicates the difference between IGF-I TAD SDS and IGF-I_avg_ SDS as predicted by the formulas for IGF-I_avg_ by time after dose for the 4 non-GHD indications (SGA, ISS, NS, and TS). The dotted gray curve indicates the difference between IGF-I TAD SDS and IGF-I_avg_ SDS as predicted by the formulas for IGF-I_avg_ by time after dose for the GHD indication as previously described [12]. The dotted vertical line indicates the time of maximum and minimum IGF-I SDS concentrations. Abbreviations: GHD, growth hormone deficiency; IGF-I, insulin-like growth factor-I; IGF-I_avg_, average IGF-I in a dosing interval; ISS, idiopathic short stature; NS, Noonan syndrome; SDS, standard deviation score; SGA, small for gestational age; TS, Turner syndrome.

Linear models were established between individual estimated steady-state IGF-I SDS at different times after dosing (IGF-I_TAD_ SDS) and weekly IGF-I_avg_ concentration SDS at steady state in 292 participants born SGA or with ISS, NS or TS receiving 0.24 mg/kg/week somapacitan for 52 weeks in REAL8 and REAL9. A comparison of the observed and predicted values was enabled as visualized for IGF-I SDS over time across indications (SGA, ISS, NS, or TS) and compared to the previously described comparison for GHD [12] (Fig. 2).

**Figure 3:**
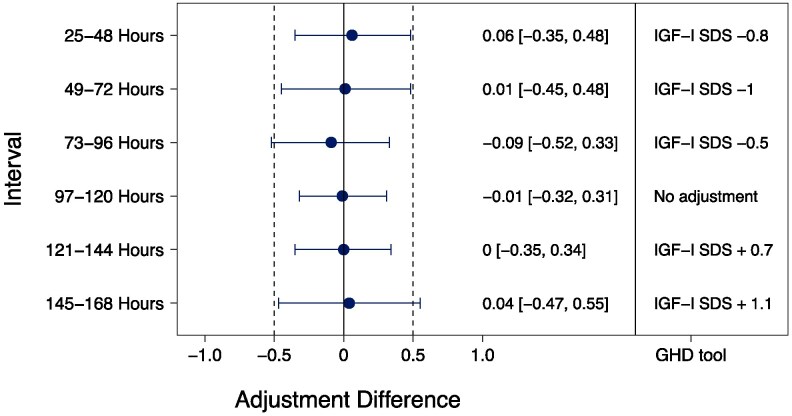
Forest plot of the model-based IGF-I_avg_ SDS by time after dose in children born SGA or with NS, TS, or ISS combined. Note: The plot shows the adjustment difference between the non-GHD indication and the previously published tool for children with GHD. The points and bars show estimated means and 90% confidence intervals for the difference to the reference (GHD tool) [12]. Vertical dotted lines indicate the [−0.5; 0.5] interval. Abbreviations: GHD, growth hormone deficiency; IGF-I, insulin-like growth factor-I; IGF-I_avg_, average IGF-I in a dosing interval; ISS, idiopathic short stature; NS, Noonan syndrome; SDS, standard deviation score; SGA, small for gestational age; TS, Turner syndrome.

Formulas were derived to allow the approximation of IGF-I_avg_ SDS based on IGF-I SDS measurements on different days within the weekly somapacitan dosing interval and then compared to the previously published tool for children with GHD [12]. The IGF-I value closest to the IGF-I average occurred approximately 4 days (97-120 hours), and the adjustment to IGF-I values and IGF-I SDS recommended for children with GHD remain relevant to adolescents and children born SGA or with ISS, NS, or TS (Fig. 3).

### Dose-response relationships for IGF-I

**Figure 4:**
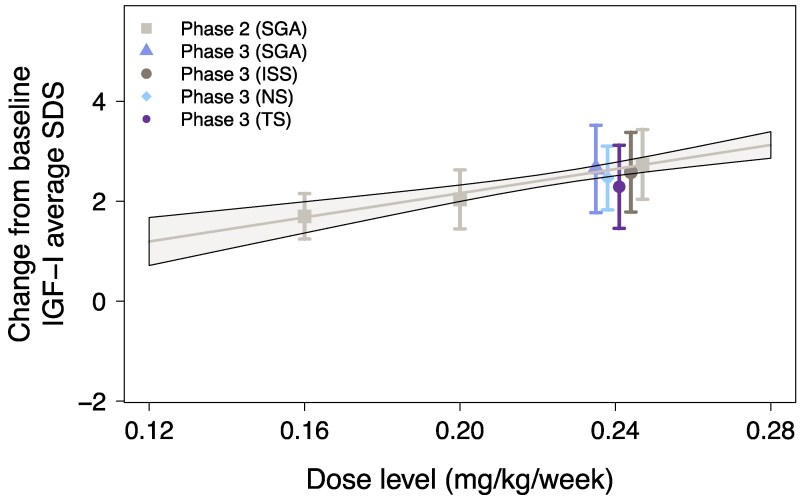
Somapacitan dose and change in IGF-I average SDS relationship in prepubertal short children born SGA or with ISS, NS, or TS. Note: Points + bars are mean + standard deviation of change from baseline in IGF-I_avg_ SDS of the dose group. The line is a linear fit to individual estimates with 95% confidence intervals. 0.16 and 0.20 mg/kg doses were only available for children born SGA. The data points at 0.24 mg/kg/week are plotted with slight separation on the x-axis for visual purposes. Abbreviations: IGF-I, insulin-like growth factor-I; IGF-I_avg_, average IGF-I over a dosing interval; ISS, idiopathic short stature; NS, Noonan syndrome; SDS, standard deviation score; SGA, small for gestational age; TS, Turner syndrome.

**Figure 5:**
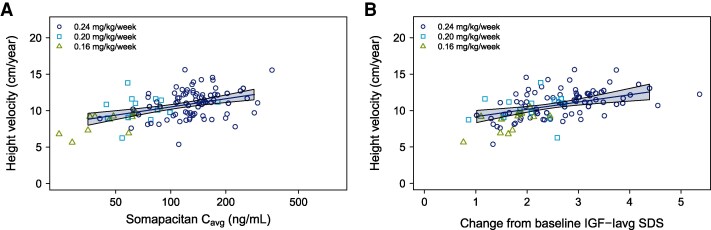
Height velocity (cm/year) vs somapacitan exposure and vs average change from baseline IGF-I response in prepubertal short children born SGA (studies REAL5 and REAL8) (panels A, B). Note: Open shapes are individual, observed height velocities vs (A) C_avg_, and (B) change from baseline in IGF-I_avg_ SDS. Triangles are somapacitan 0.16 mg/kg/week, squares are 0.20 mg/kg/week, and circles are 0.24 mg/kg/week. Lines with shaded areas are population predicted exposure-response relationships with 95% CIs from model fit to subject-level data. Abbreviations: IGF-I, insulin-like growth factor-I; IGF-I_avg_, average IGF-I in a dosing interval; SDS, standard deviation score; SGA, small for gestational age.

The dose-response relationships between somapacitan and the change in baseline IGF-I_avg_ SDS in children born SGA were well characterized by a linear model (gray line, Fig. 4): a change in somapacitan dose of 0.02 mg/kg/week in the participants’ born SGA was estimated to result in an average IGF-I SDS change of 0.24 [0.15;0.33]_95%CI_. Specific dose-response relationships could not be directly evaluated in children with ISS, NS or TS as only one somapacitan dose was evaluated in these populations (Fig. 4). In children born SGA, as well as those with ISS, NS, or TS, a similar change in baseline IGF-I_avg_ SDS was observed. Furthermore, similar exposure–response relationships between somapacitan exposure and HV (Fig. 5A) as well as between the change from baseline IGF-I_avg_ SDS and HV (Fig. 5B) observed in all participants, supporting a comparable dose–response relationship to children born SGA may be likely across the study populations.

### Exposure-response relationship between somapacitan exposure and HV for SGA

Significant exposure–response relationships between somapacitan exposure and HV (Fig. 5A) as well as between the change from baseline IGF-I_avg_ SDS and HV (Fig. 5B) were observed after 52 weeks across the 3 doses evaluated in studies REAL5 and REAL8 in participants born SGA. Similar exposure–response analyses are not presented in participants with ISS, NS, or TS, as only one somapacitan (0.24 mg/kg/week) was investigated in study REAL8.

### IGF-I response in somapacitan dosing adjustments

**Figure 6:**
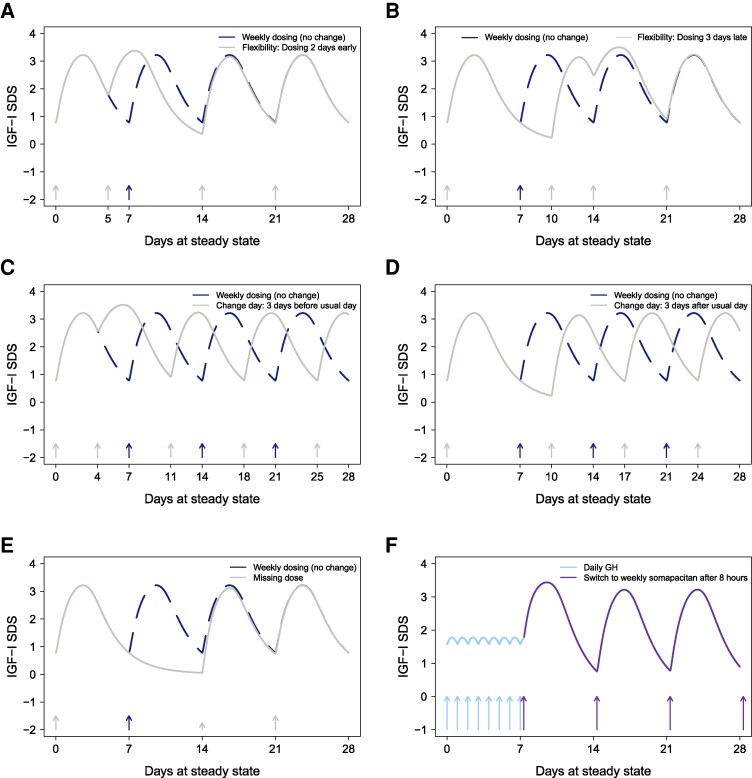
Simulations support a change of dosing day for children born SGA. Note: Lines are means of individual predictions. The gray solid line represents the actual profile, whereas the blue dotted line represents the profile had the change not occurred. Dotted lines represent the regular dosing interval. A: Dosing 2 days early; B: Dosing 3 days late; C: Changing dosing 3 days before the usual dosing day; D: Changing dosing 3 days after the usual dosing day; E: Missing a dose; F: Switch to weekly somapacitan after 8 hours. Abbreviations: GH, growth hormone; IGF-I, insulin-like growth factor-I; ISS, idiopathic short stature; NS, Noonan syndrome; SDS, standard deviation score; SGA, small for gestational age; TS, Turner syndrome.

Simulated IGF-I SDS profiles demonstrated the potential impact of dosing flexibility and scheduling changes. For children born SGA, the effect of these alterations was found to be minor across all groups, with observed increases in PK peak of ≤9.5% and IGF-I SDS changes of ≤0.55 SDS. Steady state was achieved 1-2 doses after resuming regular weekly treatment (Fig. 6). For children with ISS, similar allowances were assessed, resulting in PK peak increases of ≤10.5% and IGF-I SDS changes of ≤0.56 SDS, with steady-state reached after 1-2 doses (Fig. S1 [15]). For children with NS, the changes were again minor, with PK peak increases of ≤12.3% and IGF-I SDS changes of ≤0.54 SDS, with steady state reached after 1-2 doses (Fig. S2 [15]). For children with TS, the changes were again minor, with PK peak increases of ≤5.9% and IGF-I SDS changes of ≤0.44 SDS, with steady state reached after 1-2 doses (Fig. S3 [15]).

Additionally, for each indication, the IGF-I SDS profile was simulated when switching from daily GH to once-weekly somapacitan 8 hours after the latest daily dose. For SGA, the maximum IGF-I SDS for the first somapacitan dose after the switch was predicted to be 0.22 SDS higher than the steady state profile, for ISS, it was predicted to be 0.27 SDS higher, for NS it was predicted to be 0.18 higher, and for TS, it was predicted to be 0.67 SDS higher, with steady state reached after the second somapacitan dose.

In conclusion, the simulations revealed that changes in dosing schedules, such as flexibility in administration timing and missed doses, had a minimal impact on the PK and IGF-I profiles across all groups, consistent with observations in children with GHD. Examples are shown for SGA for dosing 2 days early or 3 days late but returning to the usual dosing day, as well as moving the dosing day 3 days earlier and later than the usual dosing day (Fig. 6). Similar results were observed (Figs. S1-S3 [15]) for children with ISS, NS, or TS, respectively.

## Discussion

The analysis presented here offers new information in relation to the use of somapacitan and practical guidance for monitoring IGF-I concentrations in children born SGA or with ISS, NS, or TS. This builds on the findings from phase 2 (REAL5 in children with SGA) [10] and phase 3 (REAL8 and REAL9) clinical studies, conducted in children born SGA (*N* = 139) or with ISS (*N* = 69), NS (*N* = 62), or TS (*N* = 79). In essence, the data support the inclusion of the 4 listed conditions as approved indications for somapacitan in children and adolescents with short stature. The findings on expected weekly average IGF-I concentrations and insights on dosing initiation and dosing day flexibility all align with prior recommendations regarding somapacitan for children with GHD [12].

The linear relationship between somapacitan dose and change in baseline IGF-I in children born SGA, as observed previously in children with GHD, was affirmed [12]. Dose change of 0.02 mg/kg/week resulted in a mean IGF-I SDS change of 0.24 [0.15;0.33]_95% CI_. This highlights the clinically relevant overall predictability of the estimated impact of reducing or increasing the somapacitan dose. While dose–response relationships could not be directly assessed in ISS, NS and TS due to the single dosage used (0.24 mg/kg/week) in the studies, the findings for SGA provide a proxy for the expected responses in these other indications given the similar change from baseline IGF-I SDS. In comparison, we previously showed that a similar dose change of 0.02 mg/kg/week resulted in a mean IGF-I SDS change of 0.32 [0.28; 0.37]_95% CI_ in children with GHD [16-18].

Consistent relationships were observed for the SGA indication between somapacitan exposure, change from baseline IGF-I and HV with increasing doses of somapacitan. These results reassure that the dose–response relationship is well understood, and that the effects of varying the dose of somapacitan can be predicted reliably. Although similar analyses were not available for ISS, NS, or TS, the similarity of the presented data across all indications (including previous findings for GHD) suggests that similar relationships may exist for these other conditions as well.

Body weight was confirmed as the major driver of somapacitan exposure across all indications, consistent with previous observations for somapacitan in GHD [12], while demographic factors such as sex and race showed minimal effect. This highlights the importance of using a weight-based dosing strategy (mg/kg/week) to optimize treatment. This is consistent with other marketed LAGH compounds [19-21].

Based on these analyses, the IGF-I value closest to the IGF-I average occurred approximately 4 days (97-120 hours) after dosing. For samples collected outside the average IGF-I time point, linear models enabled the evaluation of correction factors, which supplement the guidance provided in the somapacitan label [16-18]. The estimated correction factors fall within the confidence intervals of the existing table for somapacitan in GHD [16-18]. In other words, following somapacitan treatment, the same adjustments as outlined for GHD are also applicable across all weekly time intervals for the SGA, ISS, NS, and TS.

Once-weekly somapacitan should be administered on the same day each week. However, as this is not always possible, it is of value to assess the impact of changes in the actual dosing day, Simulation analyses focused on the effects of dosing variability, showed that dosing within a range of 3 days before or after the usual dosing day, missing a dose, or pre- or postponing dosing days by up to 3 days resulted in negligible changes in both PK profiles and IGF-I concentrations. Specifically, observed increases in PK peak were limited to a maximum of 12.3%, with IGF-I SDS changes recorded at less than 0.56 SDS across the tested indications. Importantly, after one or 2 doses of somapacitan, the steady-state concentrations were regained, further demonstrating the consistency in the treatment effect despite dosing flexibility.

In children with ISS, NS, or TS, only one somapacitan dose was evaluated and therefore the dose-response relationship could not be characterized in these populations. The analysis is based on data emerging from an RCT setting: in real-world settings, additional factors (eg, lower treatment adherence) may impact IGF-I levels and treatment outcomes. The somapacitan dose should always be individualized based on all available data, including when the last dose was administered and how adherent the patient may have been.

Finally, for children transitioning from daily GH treatment, the results posited that switching to once-weekly somapacitan yielded a maximum IGF-I SDS increase of approximately 0.27 for ISS and NS, 0.22 for SGA, and 0.67 for TS during the transition, indicating effective maintenance of therapeutic concentrations across all populations.

## Conclusion

In the current analysis, we present important insights for once-weekly somapacitan dosing and IGF-I monitoring across short children born SGA or with ISS, NS, or TS. A linear dose–response relationship was observed between somapacitan dose and change in baseline IGF-I_avg_ SDS in children with SGA; a similar relationship is anticipated in children born with ISS, NS or TS. Consistent with what has been reported in children with GHD receiving somapacitan, our findings support dosing flexibility, with changes of up to 3 days before or after the regular dosing day having a negligible impact on the IGF-I SDS profile; steady state is expected to re-establish within 1-2 doses. Our results also support the use of the previously published IGF-I calculation formulas for GHD in these new indications. These correction factors enable the consideration of available IGF-I data, allowing healthcare providers to refine and individualize their treatment strategies.

## Data Availability

Original data generated and analyzed during this study are included in this published article or in the data repositories listed in References.

## Clinical trial information

REAL8 (NCT05330325), REAL9: (NCT05723835), REAL5 (NCT03878446) and NCT01973244.

## Abbreviations

GH: growth hormone
GHD: growth hormone deficiency
IGF-1: insulin-like growth factor I
ISS: idiopathic short stature
LAGH: long-acting growth hormone
LLOQ: lower limit of quantification
NS: Noonan syndrome
PK/PD: pharmacokinetic/pharmacodynamic
SDS: standard deviation score
SGA: small for gestational age
TAD: times after dose
TS: Turner syndrome
